# Could COVID‐19 Infect the Consumer Prices Index?*

**DOI:** 10.1111/1475-5890.12229

**Published:** 2020-07-06

**Authors:** Richard Blundell, Rachel Griffith, Peter Levell, Martin O'Connell

**Affiliations:** ^1^ University College London; Institute for Fiscal Studies; ^2^ University of Manchester; Institute for Fiscal Studies; ^3^ Institute for Fiscal Studies; ^4^ Institute for Fiscal Studies

**Keywords:** inflation, COVID‐19

## Abstract

The spread of COVID‐19 has led to sweeping changes in the way households work, spend their time and shop, resulting in different shopping patterns and rapid price changes in some goods. How will changes such as these be reflected in headline inflation measures such as the Consumer Prices Index (CPI)? This paper discusses problems in interpreting the CPI as a measure of how the cost of living is changing during the lockdown.

The spread of COVID‐19 has led to sweeping changes in the way households work, spend their time and shop. This has led to large changes in spending patterns and, in some cases, rapid price changes. Web‐scraped data collected by the Office for National Statistics (ONS) indicate that online prices of cough and cold medication shot up by 11 per cent between the third and fourth weeks of March.^1^ Prices across all ‘high‐demand products’, including medicines but also nappies, pet food and rice (among others), rose by 1.1 per cent in that week. These changes could reflect increased profit margins, but also increases in firms’ costs due to the disruption that lockdown caused to supply chains and production.

How will changes such as these be reflected in headline inflation measures such as the Consumer Prices Index (CPI)? The CPI aims to measure changes in the costs of goods and services households consume. It is used extensively both in and outside government – for example, in wage negotiations and to uprate many taxes, benefits and public sector pensions. It is also the measure of inflation targeted by the Monetary Policy Committee of the Bank of England.

To calculate the CPI, the ONS collects the prices of over 700 ‘representative items’ in different regions and types of shops across the UK. The list of items is updated each year. Price collectors survey the prices of examples of these items each month to see how they are changing, holding constant features such as brand, make and pack size. Average price changes for each item are then weighted according to their importance in households’ budgets in a base year.

In normal times, the CPI measures how much the cost of purchasing a typical ‘basket’ of goods and services has changed over time, giving us a reasonable idea of how price increases are affecting households, or at least a ‘typical’ one.

However, we are not living in normal times. Some of the problems caused by the spread of COVID‐19 are logistical. The ONS now faces severe challenges in collecting prices for certain goods and services. Social distancing measures make it harder to do in‐person surveys; many shops have also closed, and some items are no longer stocked. More importantly, however, there are additional problems in *interpreting* the CPI as a measure of how the cost of living is changing, even setting these measurement issues aside.

Four particular issues stand out:

- **The basket of items on which the CPI is based will no longer be representative of actual spending**. For example, meals out, package holidays, and recreational and cultural services have weights of 9.9 per cent, 4.2 per cent and 3.4 per cent in the 2020 CPI.^2^ Yet none are currently available. Even if the prices of such items could be easily collected in the coming months, they are clearly going to be far less relevant for households currently than they are normally.
- **The CPI basket for 2021 and 2022 would normally be based on spending taking place in 2020**.^3^ This is likely to be highly unrepresentative of spending in coming years, so the problems with interpreting the CPI will extend beyond 2020. Possible solutions, if we think that changes in purchasing behaviour are only temporary, would be to disregard spending data from 2020 when constructing weights for future years – for example, use spending patterns from 2019 instead – or to predict what spending patterns across different items would have been in 2020 in the absence of social distancing.
- **Some cost increases may not be recorded**. Supply disruptions and stockpiling are forcing consumers to switch to smaller (and higher‐cost‐per‐unit) pack sizes, or to different brands from the one they typically buy. People may also have to shop in different stores where the prices of the goods they buy are higher. Because the CPI tracks the prices of fixed items in fixed locations, these extra costs will not be reflected in headline inflation. What is more, the ONS disregards multi‐buy discounts (such as buy‐one‐get‐one‐free offers) when collecting price quotes. If the prevalence of such offers does not change much over time, this does not matter much for the measurement of inflation. However, it looks as if such discounts have been abruptly halted by many supermarkets.^4^ This change will not be registered in the ONS's price survey, and price increases for certain goods will be understated.
- **The gap between what the CPI is actually measuring – the increase in the prices of goods and services – and what it is often thought to be measuring – the increase in the cost of maintaining a particular standard of living – will be much bigger than usual**. Consumers face reduced choice. Certain goods are disappearing from shelves and many services are no longer being provided. For example, consumers cannot, for the time being, enjoy restaurant meals or trips to the cinema. This has consequences for consumer welfare that the CPI will not reflect (in the same way it does not reflect the benefits that flow from the introduction of new items).

These same issues, of course, also apply to other inflation measures such as the Retail Prices Index (which is used to set price caps for utilities and to calculate the interest rates on inflation‐indexed government gilts).

The importance of these problems will depend on how long social distancing measures last and how long‐lasting the impact is on people's spending patterns. If disruption is short‐lived, and consumers’ spending patterns return to normal soon, then annual measures of inflation will not be affected much. Spending on package holidays may, for example, just be postponed until later in the year, and so its 4.2 per cent weight in the basket will be a reasonable approximation of typical expenditure. Month‐to‐month inflation rates may over‐ or under‐state price changes, but the annual cost of purchasing the CPI basket of goods would remain a reasonable benchmark for setting wages, for example.

However, if – as seems likely – disruptions last for longer, or if they have long‐lasting impacts on people's behaviour, this would lead to greater problems in interpreting the CPI. For example, if people travel less for holidays over the coming years, then the 4.2 per cent weight for package holiday spending may no longer be representative. The standard way that the weights of items in the representative basket are revised will not pick up these changes for up to two years, meaning that the CPI will no longer reflect changes in the prices of the basket even of the ‘typical’ person.

When it comes to measuring current hardship, even short‐term deviations between the rate of inflation that the CPI measures and the rate of inflation that households truly face will matter a great deal. For example, we might be particularly concerned if the nature of price rises means that lower‐income households, which are in general less able to weather short‐term financial shocks, face a bigger squeeze on their incomes than headline inflation rates suggest.

Some of the issues we have mentioned may be less of a problem for poorer households. For example, reductions in product variety or opportunities to eat out may be more relevant for the rich. However, other factors might lead the CPI to understate cost‐of‐living increases for poorer households in particular. Lower‐income households may be more affected by under‐recorded price increases, as they are more likely to purchase larger pack sizes as a way to reduce their shopping costs.^5^ Poorer households also tend to spend less on holidays and eating out than the average household, and so if this year's CPI over‐weights these items, it will be even less representative of their inflation experiences than it is normally.^6^

In addition, some monthly CPI values are more important for policy purposes than others. For example, the September CPI in each year is used to uprate taxes and benefits the following April. A key issue will therefore be the extent to which this is representative of the price changes that people have faced over the course of the year.

All of the problems we have discussed affect interpretation of the CPI in normal times as well. The current crisis has simply brought these issues to the fore, and users of the CPI will therefore need to take extra care when deciding how to apply it. More detailed real‐time data on both prices and spending patterns could help to resolve some of these problems in future, and also allow cost‐of‐living indices to be published for different income groups and household types.

## Footnotes

1 Office for National Statistics, 2020a.

2 Office for National Statistics, 2020b, table C.

3 The weights for subclasses and higher levels of aggregation in the CPI are taken from the household final consumption expenditures in the National Accounts from two years ago (as these are published with a lag). Item‐level weights are derived from more up‐to‐date sources. See Office for National Statistics (2019).

4 According to media reports, multi‐buy offers were eliminated at Tesco, while Waitrose removed 900 multi‐buy offers. All major supermarkets sharply reduced the number of promotions. (Nott, 2020.)

5 Griffith et al. (2009) estimate that savings from bulk‐buying were 2 percentage points higher relative to total shopping costs for households with an annual income under £20,000 than they were for those with an income greater than £70,000. The same paper shows that savings from multi‐buy discounts were greatest for middle‐income households, i.e. those with incomes between £30,000 and £40,000. Poorer households spend relatively more of their budgets on groceries such as food generally and so may well be more affected by the end of multi‐buy offers in supermarkets even if they do not use these offers as intensively.

6 The extent to which the unavailability of these items brings the CPI closer to or further away from the inflation experiences of poorer households depends on how the prices of these items are measured in practice. During the lockdown, the ONS has been imputing the prices of ‘unavailable items’ using price changes for the all‐items index, i.e. for prices of goods and services that are available (Office for National Statistics, 2020c). Since available items are more likely to be necessities or essentials, this could make the index *more* representative of poorer households’ spending, at least for the months when other goods are unavailable.
